# Improved Characteristics of RANKL Immuno-PET Imaging Using Radiolabeled Antibody Fab Fragments

**DOI:** 10.3390/pharmaceutics14050939

**Published:** 2022-04-26

**Authors:** Jonatan Dewulf, Ivanna Hrynchak, Sarah Geudens, Isabel Pintelon, Christel Vangestel, José Sereno, Peter A. van Dam, Antero J. Abrunhosa, Filipe Elvas, Tim Van den Wyngaert

**Affiliations:** 1 Molecular Imaging Center Antwerp (MICA), Integrated Personalized and Precision Oncology Network (IPPON), Faculty of Medicine and Health Sciences, University of Antwerp, Universiteitsplein 1, Wilrijk, B-2610 Antwerpen, Belgium; jonatan.dewulf@uantwerpen.be (J.D.); sarah.geudens@telenet.be (S.G.); christel.vangestel@uantwerpen.be (C.V.); 2 ICNAS-Produção Unipessoal Lda., Pólo das Ciências da Saúde, University of Coimbra, Azinhaga de Santa Comba, 3000-548 Coimbra, Portugal; ivanna.ua@icnas.uc.pt (I.H.); antero@pet.uc.pt (A.J.A.); 3 Laboratory of Cell Biology and Histology, Faculty of Pharmaceutical, Biomedical and Veterinary Sciences, University of Antwerp, Universiteitsplein 1, Wilrijk, B-2610 Antwerpen, Belgium; isabel.pintelon@uantwerpen.be; 4 Department of Nuclear Medicine, Antwerp University Hospital, Drie Eikenstraat 655, B-2650 Edegem, Belgium; 5 Institute for Nuclear Sciences Applied to Health (ICNAS/CIBIT), Pólo das Ciências da Saúde, University of Coimbra, Azinhaga de Santa Comba, 3000-548 Coimbra, Portugal; josesereno@uc.pt; 6 Multidisciplinary Oncologic Centre Antwerp (MOCA), Integrated Personalized and Precision Oncology Network (IPPON), Antwerp University Hospital, Drie Eikenstraat 655, B-2650 Edegem, Belgium; peter.vandam@uza.be

**Keywords:** RANKL, antibody, Fab fragment, tumor imaging, immuno-PET

## Abstract

Purpose: RANKL expression in the tumor microenvironment has been identified as a biomarker of immune suppression, negating the effect of some cancer immunotherapies. Previously we had developed a radiotracer based on the FDA-approved RANKL-specific antibody denosumab, [^89^Zr]Zr-DFO-denosumab, enabling successful immuno-PET imaging. Radiolabeled denosumab, however, showed long blood circulation and delayed tumor uptake, potentially limiting its applications. Here we aimed to develop a smaller radiolabeled denosumab fragment, [^64^Cu]Cu-NOTA-denos-Fab, that would ideally show faster tumor accumulation and better diffusion into the tumor for the visualization of RANKL. Experimental design: Fab fragments were prepared from denosumab using papain and conjugated to a NOTA chelator for radiolabeling with ^64^Cu. The bioconjugates were characterized in vitro using SDS-PAGE analysis, and the binding affinity was assessed using a radiotracer cell binding assay. Small animal PET imaging evaluated tumor targeting and biodistribution in transduced RANKL-ME-180 xenografts. Results: The radiolabeling yield of [^64^Cu]Cu-NOTA-denos-Fab was 58 ± 9.2%, with a specific activity of 0.79 ± 0.11 MBq/µg (*n* = 3). A radiotracer binding assay proved specific targeting of RANKL in vitro. PET imaging showed fast blood clearance and high tumor accumulation as early as 1 h p.i. (2.14 ± 0.21% ID/mL), which peaked at 5 h p.i. (2.72 ± 0.61% ID/mL). In contrast, [^64^Cu]Cu-NOTA-denosumab reached its highest tumor uptake at 24 h p.i. (6.88 ± 1.12% ID/mL). [^64^Cu]Cu-NOTA-denos-Fab specifically targeted human RANKL in transduced ME-180 xenografts compared with the blocking group and negative ME-180 xenograft model. Histological analysis confirmed RANKL expression in RANKL-ME-180 xenografts. Conclusions: Here, we report on a novel RANKL PET imaging agent, [^64^Cu]Cu-NOTA-denos-Fab, that allows for fast tumor imaging with improved imaging contrast when compared with its antibody counterpart, showing promise as a potential PET RANKL imaging tool for future clinical applications.

## 1. Introduction

The receptor activator of nuclear factor kappa B ligand (RANKL) is a transmembrane type 2 protein from the tumor necrosis factor (TNF) superfamily. Under physiological conditions, the predominant role of RANKL and its receptor RANK is the regulation of bone remodeling—namely, the initiation of osteoclast formation and activation [1]. RANKL is also present in epithelial and stromal components of the mammary gland, thymus, liver, and prostate. Furthermore, RANK/RANKL signaling is crucial for mammary gland development and functional lymph nodes [2,3]. However, this pathway has also been identified as an important component in carcinogenesis, specifically in up-regulating anti-apoptotic pathways and maintaining the self-renewal of cancer stem cells [4]. More recently, RANKL has been studied as an emerging target in the tumor immune intrinsic crosstalk, where RANKL overexpression can negate the effect of immunotherapies [5,6]. Gomez-Aleza et al. showed in their clinical and pre-clinical work that denosumab (a human IgG2 monoclonal anti-RANKL antibody) treatment could convert a tumor hostile to immune-mediated therapies in an immune-susceptible environment [7].

Unfortunately, RANKL assessment in patients suffers from inadequate assessment techniques due to the considerable target heterogeneity and imperfect serum analysis methods [8]. Therefore, its role in the tumor microenvironment (TME) of cancer patients is not entirely understood. The use of non-invasive molecular imaging techniques such as positron emission tomography (PET) offers substantial advantages over tissue sampling. It provides a separate assessment of the primary and metastatic tumor sites within a single scan, allowing spatial quantification of tumor heterogeneity and phenotypic discordance between tumor sites [9,10]. Additionally, non-invasive alternatives (so-called liquid biopsies) that rely on circulating tumor cells or tumor-shed products may not always reliably reflect the heterogeneity of the primary tumor or the dominant disease bulk, and standardization of these assays has been difficult in clinical practice [11,12,13]. Finally, the non-invasive nature of PET imaging has been reported to reduce patient stress levels, anxiety, and pain [14].

ImmunoPET is an emerging imaging technique that uses radiolabeled monoclonal antibodies (mAbs) to assess the expression of specific markers non-invasively. Previously, we had successfully developed ^89^Zr-radiolabeled denosumab, which targets and binds huRANKL with high affinity and specificity for immuno-PET of RANKL. In vivo, [^89^Zr]Zr-DFO-denosumab PET imaging demonstrated, in RANKL transduced xenografts, a peak uptake of 26% ID/g at 5 days post injection (p.i.). However, high blood pool radioactivity could be observed for multiple days post injection, indicating slow biodistribution that resulted in poor tumor contrast at early time frames [15]. Additionally, the long half-life of ^89^Zr (t_1/2_ = 78.41 h) and high energy gamma emission (908.97 keV) of ^89^Zr can result in a high radiation dose [16].

Antibodies are unmatched in target specificity and affinity, but their high molecular weight can be a limiting factor for optimal tumor targeting. Vascular permeability, tumor diffusion, and tumor penetration are all inversely correlated with the size of these macromolecules [17,18]. Additionally, most solid tumors exhibit an enhanced permeability and retention (EPR) effect that contributes to increased non-specific tumor uptake [19,20].

Several approaches have been developed successfully to overcome these limitations, such as the use of antibody fragments, nanobodies, and a pretargeting approach [21,22]. Therefore, we propose to improve the translational potential of our RANKL imaging agent by developing a lower-molecular weight radiolabeled antibody fragment (Fab)—[^64^Cu]Cu-NOTA-denos-Fab—which has intrinsic shorter circulation times, uses short half-lived radiometals, and combines fast renal clearance with improved distribution throughout the tumor [23].

## 2. Materials and Methods

All products were obtained from Sigma-Aldrich (St. Louis, MO, USA) unless stated otherwise.

### 2.1. Fab Fragment Preparation and Purification

Denosumab (AMG162; human antibody; IgG2; denosumab [XGEVA], Amgen Inc., Thousand Oaks, CA, USA), targeting human RANKL, was purified and buffer exchanged to phosphate-buffered saline (PBS, 0.01 M, pH 7.4) from the manufacturer’s formulation solution using a PD-10 column size exclusion column (Cytiva, Marlborough, MA, USA) before further use. Immobilized papain resin (Thermo Fisher Scientific, Waltham, MA, USA) was pre-incubated, activated, and washed 3 times using 80 mM cysteine in PBS. The digestion was performed by adding 5 mg AMG162 to the immobilized papain resin (1/10 ratio) in 80 mM cysteine in PBS solution, followed by incubation for 24 h at 37 °C under continuous stirring. The digestion efficiency (% area under the curve = $\frac{Fab\;\&\;Fc\;fragments}{Partially\;\&\;undigested\;Antibody\;+\;fab\;\&\;Fc\;fragments}$) was evaluated using size exclusion-high pressure liquid chromatography (SEC-HPLC) (Superdex 200 increase 5/150, 0.01 M PBS pH 7.4, flowrate: 0.15 mL/min, λ = 280 nm, Cytiva).

Before purification, the digestion mixture was concentrated and buffer exchanged (50 mM phosphate buffer, pH 8) using a centrifugal filter unit (30 kDa cut-off, Sartorius). The concentrated, digested sample was then loaded onto prepacked Fabsorbent columns (F1P HF P6HF adsorbent, Astrea, Stoughton, MA, USA) and left to interact with the resin for 3–5 min. After the incubation period, the purified fractions were eluted with 0.1 M sodium phosphate/0.05 M citric acid at different pH (7, 6, 5, 4, and 3) using a flow rate of 1 mL/min for 5 min. Fab fragments in the eluted fractions were identified by SDS-PAGE and Coomassie blue staining under reducing and non-reducing conditions and compared with Fab isotype control. Eluted fractions containing Fab fragments (pH 7–4) were pooled and purified from aggregates using SEC-HPLC (Superdex 75 increase 10/300, 0.01 M PBS pH 7.4, flowrate: 0.8 mL/min, λ = 280 nm, Cytiva). Protein concentration was measured using a spectrophotometer (Genesys 10S UV-VIS) at λ = 280 nm. At least 3 different batches were pooled and used in subsequent experiments to avoid batch-to-batch variability. Different methods and strategies for the preparation and purification of Fab fragments were also explored in this research project and can be found in the Supplementary Materials (Figure S1 and Table S1).

### 2.2. Bioconjugation and Affinity Evaluation

Denosumab and the Fab fragments were conjugated via a random lysine using the bifunctional chelator p-Bn-SCN-NOTA (Macrocyclics, Plano, TX, USA). The antibody solution (3 mg/mL) and Fab fragment solution (1.5 mg/mL) were adjusted to pH 8.8–9 using sodium carbonate, and a molar excess of 5 or 10 equivalents of the chelator was added to the solutions. The reaction proceeded for 2 h at 37 °C. Afterward, the chelator was removed from the solution using a PD10 column and buffer-exchanged to chelex (100 mesh)-treated 0.1 M NH_4_OAc, pH 7. The bioconjugates were characterized by ESI-HRMS (Centre for Proteomics, University of Antwerp) using a Q-TOF2 instrument (Waters, Milford, MA, USA), as described previously [15].

The binding affinity of the native antibody and fragments, together with their NOTA-conjugated counterparts, was determined via an ELISA assay to evaluate the impact of the conjugation and antibody fragmentation. For this, soluble RANKL (Novusbio, Centennial, CO, USA, NBP1-72339, 50 nM, 100 µL) was coated to a 96-well plate and incubated overnight at 4 °C. Afterward, the plate was washed 3 times with washing buffer (0.2% Tween/0.01 M PBS pH 7.4) and blocked with 3% BSA in PBS for 1 h at room temperature (RT). The blocking solution was washed away (3 times) and denosumab, NOTA-denosumab, Fab, and NOTA-denos-Fab were added to the wells (60 µg/mL) and serial diluted (1:5) for 9 times and incubated for 1 h at RT. After washing, the secondary antibody (Abcam, Watham, MA, USA Cat# ab6759, RRID:AB_955434) (1/130,000 dilution, 2 mg/mL stock, 100 μL) was incubated for 1 h at RT. After wash (3×), the substrate (1:1 TMB substrate kit, 100 μL, Thermo Fisher Scientific) was added to the wells and incubated for 20 min at RT. The reaction was stopped by adding 1M H_2_SO_4_, and the plate was read out at 450 nm. The assay was performed in duplicate (*n =* 2). In this assay, the assumptions inherent to the Law of Mass Action were met. This assay was performed as described before [24,25]. The data were analyzed using GraphPad Prism version 9.3.1 (San Diego, CA, USA, RRID:SCR_002798) with the nonlinear regression function (specific binding—with Hill slope).

### 2.3. [^68^Ga]Ga-NOTA-Fab/Denosumab Radiolabeling and Characterization

NOTA is a good chelator for both ^68^Ga and ^64^Cu. While the goal of this study was to use ^64^Cu, matching the biological half-life of the Fab fragment (12–20 h) [26], the characterization of the bioconjugates was performed using ^68^Ga for convenience and isotope availability, creating optimized bioconjugates for future ^64^Cu radiolabeling.

[^68^Ga]Ga^3+^ was eluted from the generator (Galli Eo^TM^, IRE Elit, Fleurus, Belgium) according to manufacturer’s instructions in 0.1 M HCl, (V = 1.1 mL). NOTA-denosumab (350 µg) or NOTA-denos-Fab (50 µg) was diluted in 1 M NH_4_OAC pH 6.5 reaction buffer (traceSelect, Sigma-Aldrich) prior to the addition of 300 MBq and 150 MBq [^68^Ga]Ga^3+^ eluate, respectively. The reaction mixture was left to incubate at 40 °C for 15 min. Afterwards, the samples were purified using a PD-10 column and eluted and reformulated with 0.01 M PBS pH 7.4. Quality control of the radiolabeled conjugates was performed using SEC-HPLC ([^68^Ga]Ga-NOTA-denos-Fab: Superdex 75 increase 10/300, 0.01 M PBS pH 7.4, flowrate: 0.8 mL/min, λ = 280 nm, Cytiva; [^68^Ga]Ga-NOTA-denosumab: Superdex 200 increase 5/150, 0.01 M PBS pH 7.4, flowrate: 0.15 mL/min, λ = 280 nm, Cytiva) and iTLC (0.1M citric acid pH 5; Rf = 0: colloids; Rf = 0: [^68^Ga]Ga-NOTA-denos-Fab/[^68^Ga]Ga-NOTA-denosumab; Rf = 1 free [^68^Ga]Ga^3+^). Stability of both radiotracers was assessed in final formulation at RT and in mouse plasma at 37 °C, for 2 h (N = 2) via iTLC.

### 2.4. [^64^Cu]Cu-NOTA-Denos-Fab/Denosumab Radiolabeling and Characterization

[^64^Cu]CuCl_2_ was produced and purified as previously described [27]. Hydrochloric acid was evaporated after purification to neutralize the solution and free [^64^Cu]Cu^4+^/colloids was determined via iTLC (0.1 M citric acid pH 5; Rf = 0: colloids; Rf = 1 free [^64^Cu]Cu^4+^) prior to use. Subsequently, [^64^Cu]CuCl_2_ (~85 MBq) was diluted in 0.1M pH 5.5 NH_4_OAc (trace select, Sigma-Aldrich), and bioconjugate NOTA-denos-Fab (50 µg) or NOTA-denosumab (200 µg) was incubated for 15 min at 37 °C. After incubation, the reaction mixtures were purified using a PD10 column, eluted using sterile saline, and purity was evaluated on SEC-HPLC (Superdex 200 increase 10/300, 0.01 M PBS pH 7.4, flowrate: 0.5 mL/min, λ = 280 nm, Cytiva) and iTLC (0.1 M citric acid pH 5; Rf = 0: colloids; Rf = 0: [^64^Cu]Cu-NOTA-denos-Fab/[^64^Cu]Cu-NOTA-denosumab; Rf = 1 free [^64^Cu]Cu^4+^). Stability of both radiotracers was assessed in final formulation at RT and in mouse plasma at 37 °C, for 40 h (N = 2) via iTLC.

### 2.5. Cell Binding Study

Human cervical cancer ME-180 (CVCL_1401, ATCC) and human RANKL-transduced ME-180 cells RANKL-ME-180, previously established at Center for Oncological Research (CORE), University of Antwerp [15], were cultured in MCoy’s 5A (Invitrogen, Waltham, MA, USA) supplemented with 10 (*v*/*v*%) fetal bovine serum (FBS) (Invitrogen), 2 mM L-glutamine (Invitrogen), and 1 (m/m%) penicillin and streptomycin (Invitrogen). Cells were incubated at 37 °C under humidified conditions with 5% CO_2_. Adherent cells were harvested using 0.05% trypsin/EDTA solution.

A suspension of 0.5 × 10^6^ RANKL-ME-180 or ME-180 cells in a final volume of 0.5 mL in FBS free media was pre-equilibrated for 30 min at 37 °C prior to the start of the assay. [^64^Cu]Cu-NOTA-denos-Fab or [^64^Cu]Cu-NOTA-denosumab was added to the cell suspension at a concentration of 5 and 20 nM, followed by incubation for 2 h at 37 °C. For the blocking control, cells were pre-incubated with ×250 molar fold excess of native antibody for 1 h at 37 °C. Afterward, cells were centrifuged and washed once before gamma-counting the cell pellet and supernatants on a well counter (CRC-55t, Capintec, Florham Park, NJ, USA). Radiotracer binding is expressed as the percentage of cell-bound activity. The assay was performed in duplicate.

### 2.6. Animal Experiments

Animal experiments were approved by the national authority committee (DGAV, Portugal) (project number 012788, 14 September 2021) and were in accordance with the European Community Council Directive (2010/63/EU). Animals were housed at 22 ± 1 °C, 70% relative humidity, 12 h light–dark cycles, and access to water and food ad libitum.

Low-passage RANKL-ME-180 and ME-180 (8 × 10^6^) cells were harvested, suspended in 100 µL sterile PBS and subcutaneously injected into the right hindlimb of 5- to 7-week-old athymic female CD-1 nude mice (Charles River Laboratories, Wilmington, MA, USA, RRID:IMSR_CRL:086).

### 2.7. PET Imaging and Biodistribution Studies

Tumor-bearing mice (RANKL-ME-180 and ME-180, tumor diameter 5–11 mm—via caliper measurement) were injected with [^64^Cu]Cu-NOTA-denos-Fab (~33 µg; ~26 MBq; ~1.19 MBq/g (activity/weight animal); *n =* 4) or [^64^Cu]Cu-NOTA-denosumab (~139 µg; ~26 MBq; ~1.18 MBq/g (activity/weight animal); *n =* 4) via lateral tail vein injection. In a subset of RANKL-ME-180 tumor-bearing animals, a blocking study was performed via the intravenous injection of native denosumab (excess dose, 4 mg; *n =* 3) 2 days before the injection of [^64^Cu]Cu-NOTA-denos-Fab. Under isoflurane anesthesia, static whole-body PET images were acquired over 30 min and performed for all groups at 1 h, 5 h, and 24 h post radiotracer injection on an EasyPET.3D system (Radiation Imaging Technologies, Aveiro, Portugal). PET images were reconstructed via the List Mode–Median Root a Priori algorithm (beta = 0.15 and kernel size = 3) with voxel size (0.5 × 0.5 × 0.559) mm^3^ and 25 iterations. Subsequently, for co-registration, whole-body MRI acquisitions (2D localizer multi-slice and T2-weighted, coronal orientation) were performed on a 9.4T MR pre-clinical scanner (Bruker, Billerica, MA, USA) equipped with a standard volume Bruker coil setup (transmit/receiver 112/072 mm of inner/outer diameter).

Respiratory frequency and body temperature were kept constant and monitored continuously. Quantitative imaging processing was performed using PMOD (PMOD, v 3.6; PMOD Technologies, Zürich, Switzerland, RRID:SCR_016547), to delineate volumes of interest on PET images. The image VOI-derived percentage injected dose per mL (% ID/mL) was calculated as [total positron (β+ radioactivity) concentration in the VOI at the time of scan (kBq/mL)/total positron (β+ radioactivity) injected (kBq) × 100].

After the final acquisition time point, animals were sacrificed, and tumors and organs were harvested for well counter analysis in all groups. Afterward, tumors were snap-frozen in liquid nitrogen, embedded in OCT, and sectioned at 10 µm for histological analysis.

### 2.8. Histology

In each imaging group, non-adjacent 10 µm tumor sections were obtained at non-adjacent regions, providing a complete overview of target expression in the tumor. Frozen sections of 10 µm were thawed and fixed in 4% paraformaldehyde for 15 min, followed by rinse in 6 × 5 min in PBS. Afterward, sections were blocked with 5% donkey serum for 1 h at RT. Immediately after blocking, primary antibody incubation (anti-huRANKL; 1:500 dilution, Thermo Fisher Scientific, Cat# PA5-21951, RRID:AB_11156181) was performed overnight at 4 °C. The following day, tumor sections were washed and incubated with secondary donkey anti-rabbit cy3 (Jackson ImmunoResearch Labs, Cambridgeshire, UK, Cat# 711-165-152, RRID:AB_2307443) (1:200 dilution). The nucleus was highlighted with DAPI. For quantification, images were acquired in each section at random (spiral form) via a Nikon Eclipse Ti microscope (20×). Depending on the size of the section, 4–7 images were obtained. Image analysis was performed using ImageJ v1.53 (RRID:SCR_003070).

### 2.9. Statistical Analysis

Data are expressed as mean ± standard deviation (SD). Statistical analysis was performed using GraphPad Prism version 9.3.1 (RRID:SCR_002798). Statistical significance between different groups was analyzed by the one-way analysis of variance (ANOVA) followed by Bonferroni correction. Statistical significance between two data sets was evaluated by the unpaired two-tailed Student *t* test. Differences between groups were considered statistically significant if the *p* value was less than 0.05.

## 3. Results

### 3.1. Fab Fragment Preparation and Purification

**Figure 1:**
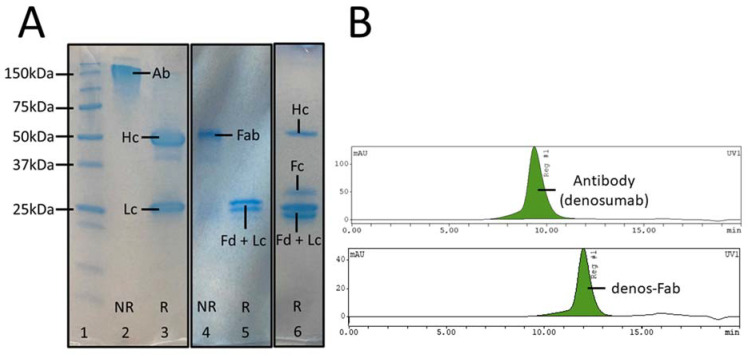
Fab fragment preparation and bioconjugation. (**A**) SDS-PAGE of denosumab. Lane 1: molecular weight markers; lane 2: non-reduced denosumab; lane 3: reduced denosumab; lane 4: non-reduced Fab fragment; lane 5: reduced Fab fragment; lane 6: reduced digested denosumab mixture. (**B**) SEC-HPLC of denosumab and purified Fab fragment (UV signal). (Ab = antibody, Hc = heavy chain, Lc = light chain, Fab = antibody Fab fragment, Fd = heavy chain of Fab fragment, Fc = crystallizable fragment region, NR = non reduced, R = reduced).

After denosumab digestion with papain undigested or partially digested antibody, Fab and Fc fragments were obtained. According to SEC-HPLC, digestion efficiency was 74 ± 10.8% (*n =* 3) after 24 h. Gradient pH elution of the Fabsorbent column showed Fab fragments eluting at pH 7–4, while Fc and intact or partially digested antibody eluted at pH 3, as identified by SDS-PAGE and SEC-HPLC (Figure 1A,B). Purified Fab fragments could be isolated with a yield of 22 ± 4.1% (*n =* 3) and >95% purity.

### 3.2. Bioconjugation and Affinity Evaluation

The conjugation efficiency of p-SCN-NOTA to the Fab fragments and antibody and the RANKL binding affinity (Kd values) for the different conjugation protocols are presented in Table 1. Fab fragments show a small increase in Kd values (lower binding affinity) compared with intact denosumab, probably due to denosumab’s multivalency, which can influence avidity. No significant difference in Kd values could be observed between native and conjugated samples of Fab and antibody. No significant difference in Kd values could be observed between the 5 eq protocol and the 10 eq conjugation protocol. NOTA-denos-Fab (10 eq) and NOTA-denosumab (5 eq) were selected for further use for radiolabeling and in vitro and in vivo studies.

### 3.3. Fab/Denosumab Radiolabeling Characterization

**Figure 2:**
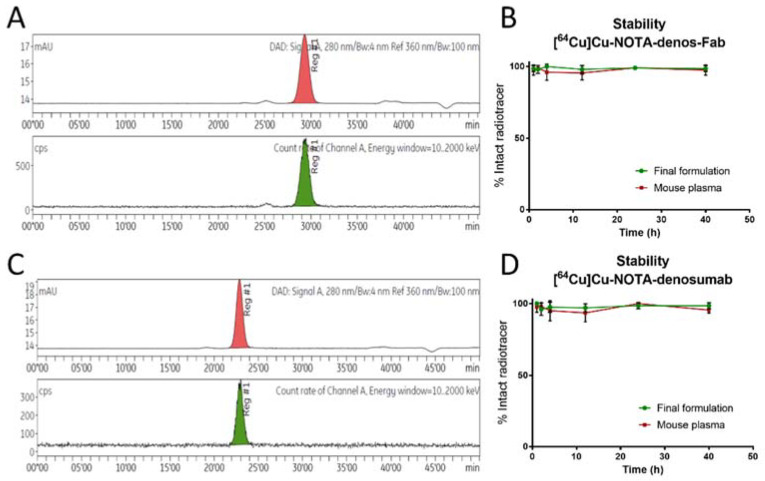
[^64^Cu]Cu-NOTA-denos-Fab/denosumab characterization. (**A**) SEC-HPLC (Superdex 200 increase 10/300) of [^64^Cu]Cu-NOTA-denos-Fab; upper part UV signal, bottom part radioactive channel. (**B**) Stability of [^64^Cu]Cu-NOTA-denos-Fab in mouse plasma and final formulation. (**C**) UV and radio-SEC-HPLC chromatograms of [^64^Cu]Cu-NOTA-denosumab after radiosynthesis; upper part UV signal, bottom part radioactive channel. (**D**) Stability of [^64^Cu]Cu-NOTA-denosumab in mouse plasma and final formulation (data graph: mean ± 1 standard deviation).

A radiolabeling overview of NOTA-denos-Fab and NOTA-denosumab can be seen in Table 2. Characterization of bioconjugates was performed with ^68^Ga and showed good apparent specific activity and stability of both radiotracers. In subsequent experiments, similar radiochemical yields (RCYs) were obtained for ^64^Cu radiolabeling, except for the lower apparent specific activity of [^64^Cu]Cu-NOTA-denosumab (Figure 2), as reported previously [28,29].

### 3.4. Cell Binding Study

**Figure 3:**
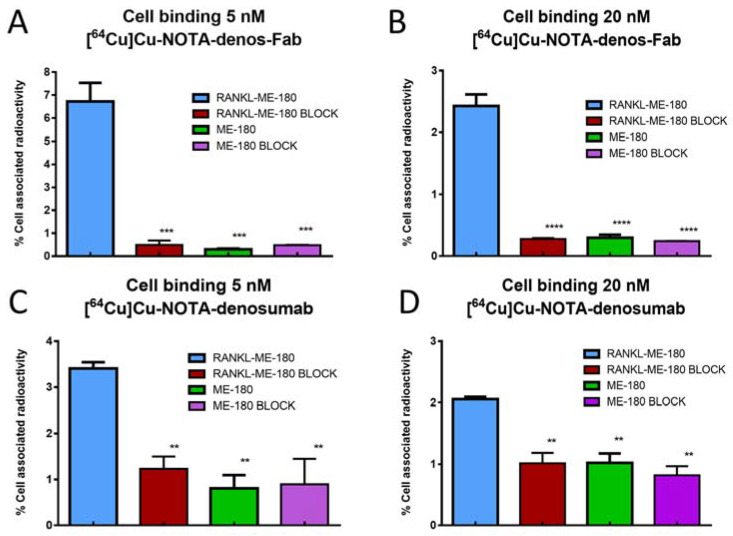
[^64^Cu]Cu-NOTA-denos-Fab/denosumab cell-binding. Binding of [^64^Cu]Cu-NOTA-denos-Fab to RANKL-ME-180 and ME-180 cells with and without blocking (mean ± 1 standard deviation; ** *p* ≤ 0.01; *** *p* ≤ 0.001; **** *p* ≤ 0.0001).

Cell binding of both radiotracers to a positive cell line (RANKL-ME-180) and negative cell line (native ME-180) was evaluated in vitro to visualize target binding and specificity. The percentage associated cell-bound activity for [^64^Cu]Cu-NOTA-denos-Fab was significantly higher on the RANKL-transduced cells (6.7 ± 0.8%) compared with the blocking control (0.5 ± 0.2%; *p =* 0.0003) and ME-180 cells (0.3 ± 0.03%; *p =* 0.0002, Figure 3A). Consecutively, at a radiotracer concentration of 20 nM, binding was also higher on RANKL-transduced cells (2.4 ± 0.2%) compared with blocking (0.3 ± 0.02%; *p* < 0.0001) and control ME-180 cells (0.3 ± 0.05%; *p* < 0.0001, Figure 3B). A reduced percentage (%) cell-bound activity was seen with higher radiotracer concentrations due to the cold mass effect, as described before [30].

[^64^Cu]Cu-NOTA-denosumab showed 3.4 ± 0.1% cell-bound associated activity compared with blocking 1.2 ± 0.3% (*p =* 0.0075) and ME-180 cells 0.8 ± 0.3% (*p =* 0.0039, Figure 3C). Similarly, at a radiotracer concentration of 20 nM, RANKL-ME-180 binding (2.1 ± 0.04%) was higher compared with blocking (1.0 ± 0.2%; *p* < 0.0001) and ME-180 cells (1.0 ± 0.2%; *p* < 0.0001, Figure 3D).

### 3.5. PET Imaging and Biodistribution Studies

**Figure 4:**
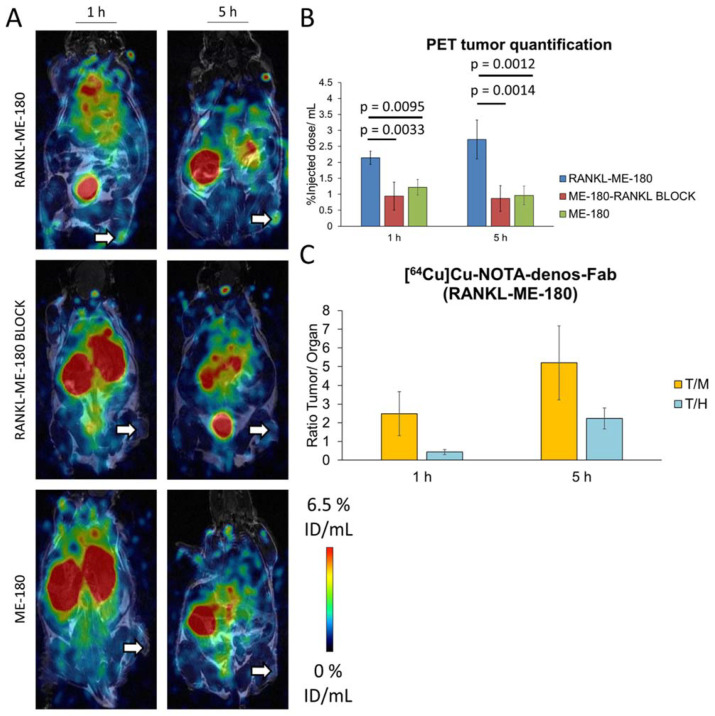
[^64^Cu]Cu-NOTA-denos-Fab PET imaging. (**A**) [^64^Cu]Cu-NOTA-denos-Fab PET images of RANKL-ME-180 (**top**), RANKL-ME-180 BLOCK (**middle**), and ME-180 (**bottom**) xenografts at 1 h and 5 h p.i. (**B**) PET analysis of [^64^Cu]Cu-NOTA-denos-Fab tumor uptake. (**C**) [^64^Cu]Cu-NOTA-denos-Fab tumor/background ratios in RANKL-ME-180 xenografts. (ID/mL: injected dose/mL; data graph: mean ± 1 standard deviation; white arrow: tumor, T/M: tumor/muscle, T/H: tumor/heart).

The in vivo targeting potential of the radiotracer [^64^Cu]Cu-NOTA-denos-Fab was assessed in RANKL-ME-180 (RANKL-positive) and ME-180 xenografts (RANKL-negative) using small animal PET imaging. [^64^Cu]Cu-NOTA-denos-Fab demonstrated a mean radiotracer uptake of 2.14 ± 0.21% ID/mL at 1 h and peaked at 5 h (2.72 ± 0.61% ID/mL) p.i. in RANKL-ME-180 xenografts. The specificity was confirmed in a blocking study with a significantly lower uptake at 1 h (0.94 ± 0.44% ID/mL, *p =* 0.0033) and at 5 h (0.87 ± 0.40% ID/mL, *p =* 0.0014) p.i. Additionally, also in negative ME-180 xenografts, [^64^Cu]Cu-NOTA-denos-Fab showed a significantly lower uptake at 1 h (1.22 ± 0.24% ID/mL, *p =* 0.0095) and 5 h (0.96 ± 0.29% ID/mL, *p =* 0.0012) p.i. At 24 h post radiotracer injection, no image acquisition was possible due to the almost complete excretion of the Fab radiotracer (Figure 4A,B).

**Figure 5:**
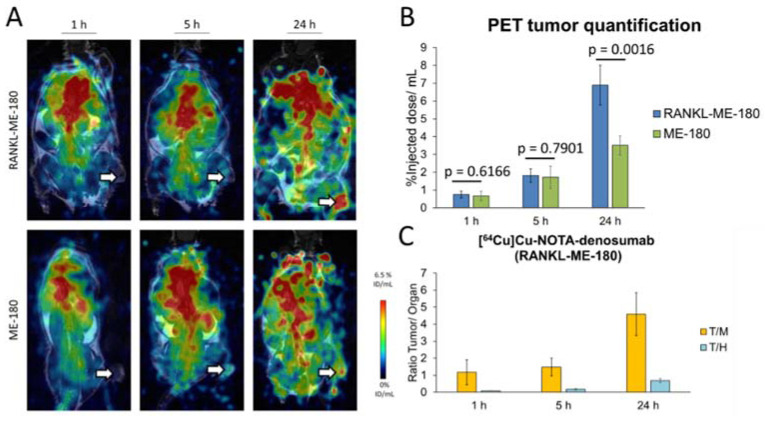
[^64^Cu]Cu-NOTA-denosumab PET imaging. (**A**) [^64^Cu]Cu-NOTA-denosumab PET images of RANKL-ME-180 (**top**) and ME-180 (**bottom**) xenografts at 1 h, 5 h, and 24 h p.i. (**B**) PET analysis of [^64^Cu]Cu-NOTA-denosumab tumor uptake. (**C**) [^64^Cu]Cu-NOTA-denosumab tumor/background ratios in RANKL-ME-180 xenografts. (ID/mL: injected dose/mL; data graph: mean ± 1 standard deviation; white arrow: tumor, T/M: tumor/muscle, T/H: tumor/heart).

To obtain a head-to-head comparison with the antibody, [^64^Cu]Cu-NOTA-denosumab was also evaluated in RANKL-ME-180 and ME-180 xenografts. In the positive model, [^64^Cu]Cu-NOTA-denosumab showed a radiotracer uptake of 0.75 ± 0.19% ID/mL at 1h, 1.82 ± 0.38% ID/mL at 5 h, and 6.88 ± 1.12% ID/mL at 24 h p.i. In the negative model radiotracer uptake was 0.67 ± 0.25% ID/mL (*p =* 0.6166) at 1 h, 1.72 ± 0.63% ID/mL (*p =* 0.7901) at 5 h, and 3.51 ± 0.54% ID/mL (*p =* 0.0016) at 24 h p.i., showing only a statistically significant difference in radiotracer uptake at the 24 h p.i. timepoint (Figure 5A,B). More importantly, [^64^Cu]Cu-NOTA-denos-Fab showed a statistically significant increase in tumor uptake compared with [^64^Cu]Cu-NOTA-denosumab at 1 h (*p =* 0.0002) and at 5 h p.i. (*p =* 0.0452) in RANKL-ME-180 xenografts.

To better understand the tumor-to-background ratios, we compared tumor-to-heart/blood (T/H = T/B) and tumor-to-muscle (T/M) ratios. Interestingly, the tumor/heart (T/H) ratio of [^64^Cu]Cu-NOTA-denos-Fab uptake at 1 h p.i. was already significantly higher compared with the value reached for [^64^Cu]Cu-NOTA-denosumab at 1 h (*p =* 0.0011), at 5 h (*p =* 0.0102), or even at 24 h (*p =* 0.0140) p.i. The T/H ratio for [^64^Cu]Cu-NOTA-denos-Fab at 5 h p.i. exceeded that of [^64^Cu]Cu-NOTA-denosumab at all time points (*p* < 0.0001). The tumor/muscle (T/M) ratio at 1 h p.i. did not significantly differ between both radiotracers. However, at 5 h after [^64^Cu]Cu-NOTA-denos-Fab injection, the T/M ratio was significantly higher compared with [^64^Cu]Cu-NOTA-denosumab (*p =* 0.0033), which reached a similar value only at 24 h p.i. Taking the aforementioned into account, we can conclude that [^64^Cu]Cu-NOTA-denos-Fab showed better tumor contrast at earlier time points compared with [^64^Cu]Cu-NOTA-denosumab (Figure 4C and Figure 5C). Maximum intensity projection (MIP) is included in the supplemental data to give the reader an overview of the radiotracer biodistribution (Supplementary Materials Figure S3).

**Figure 6:**
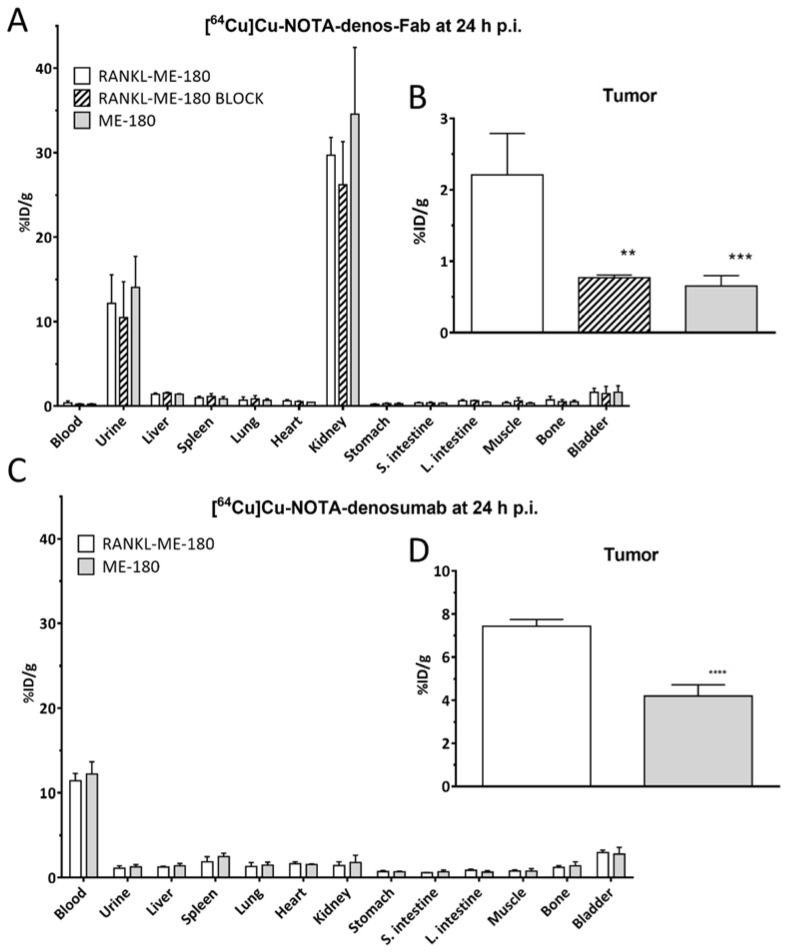
Ex vivo biodistribution analysis of [^64^Cu]Cu-NOTA-denos-Fab/denosumab. Ex vivo biodistribution analysis of [^64^Cu]Cu-NOTA-denos-Fab (**A**) in organs and (**B**) in the tumor and [^64^Cu]Cu-NOTA-denosumab (**C**) in organs and (**D**) in the tumor 24 h post radiotracer injection (mean ± 1 standard deviation; % ID/g: percentage injected dose/gram; p.i.: post injection; ** *p* ≤ 0.01; *** *p* ≤ 0.001; **** *p* ≤ 0.0001).

Ex vivo biodistribution analysis was performed after the final image acquisition at 24 h p.i. for both radiotracers. Overall, the biodistribution of [^64^Cu]Cu-NOTA-denosumab in most measured organs was higher than [^64^Cu]Cu-NOTA-denos-Fab except for the kidneys, which was consistent with the high intensity of uptake seen on the PET images. This illustrates the different excretion pathways between the antibody and the Fab fragment. Due to the smaller size of the Fab fragment compared with the antibody (50 kDa vs. 150 kDa), predominantly renal clearance of [^64^Cu]Cu-NOTA-denos-Fab (kidneys 29.70 ± 1.82% ID/g and urine 12.18 ± 2.92% ID/g) could be observed. Remarkable differences could be observed in the remaining blood pool radioactivity at 24 h between [^64^Cu]Cu-NOTA-denos-Fab (0.38 ± 0.20% ID/g) and [^64^Cu]Cu-NOTA-denosumab (11.41 ± 0.75% ID/g), indicating fast clearance for [^64^Cu]Cu-NOTA-denos-Fab compared with [^64^Cu]Cu-NOTA-denosumab (Figure 6A,C).

Ex vivo tumor uptake of [^64^Cu]Cu-NOTA-denos-Fab was significantly higher in RANKL-ME-180 xenografts (2.21 ± 0.50% ID/g) compared with blocked xenografts (0.77 ± 0.040% ID/g, *p =* 0.0016) and when compared with the negative ME-180 xenograft model (0.65 ± 0.13% ID/g, *p =* 0.006) (Figure 6B). For [^64^Cu]Cu-NOTA-denosumab, significantly higher tumor uptake could be observed between RANKL-ME-180 and ME-180 xenografts, respectively (7.43 ± 0.27 vs. 4.20 ± 0.45% ID/g, *p* < 0.0001) (Figure 6D). The tumor uptake for both radiotracers was higher than in other major organs (except clearance organs), providing a good tumor contrast. Higher T/B ratios could be observed for [^64^Cu]Cu-NOTA-denos-Fab (6.59 ± 1.58) compared with [^64^Cu]Cu-NOTA-denosumab (0.65 ± 0.048), highlighting the more favorable characteristics of [^64^Cu]Cu-NOTA-denos-Fab as a promising radiotracer with lower non-specific accumulation.

### 3.6. Histology

**Figure 7:**
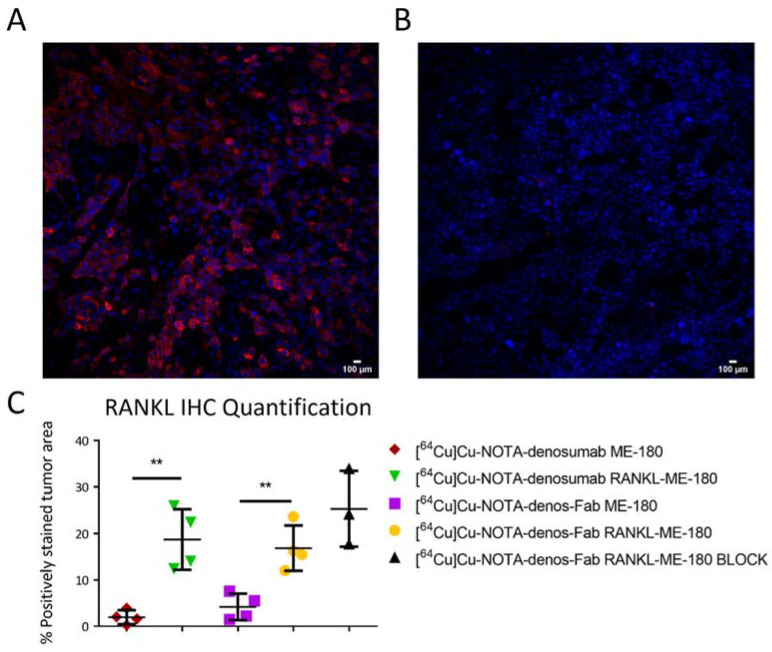
Histology and IHC quantification. Immunofluorescence staining of (**A**) RANKL-ME-180 xenografts and (**B**) ME-180 xenografts. (**C**) RANKL IHC quantification in the different animal groups. (Images acquired using 20× objective, mean ± 1 standard deviation; ** *p* ≤ 0.01).

The RANKL expression in RANKL-ME-180 and ME-180 tumor tissues was evaluated by immunofluorescent staining to corroborate the PET and ex vivo results of radiotracer uptake. RANKL-ME-180 tumors showed high levels of RANKL expression, as shown in red (Figure 7A). In contrast, RANKL expression was absent in ME-180 xenografts (Figure 7B). Quantification of RANKL expression was performed for each group of animals. As expected, RANKL positivity was higher for RANKL-ME-180 tumors when compared with ME-180 xenografts (Figure 7C).

## 4. Discussion

Denosumab was approved by the FDA nearly 15 years ago, and anti-RANKL treatment has improved clinical outcomes in patients with a wide variety of skeletal conditions (giant cell tumor of the bone, bone metastases, osteoporosis, and multiple myeloma) [31]. Moreover, in recent years the potential of denosumab has emerged to modulate immune response when combined with immune checkpoint inhibitors [32]. Ongoing clinical trials (CHARLI trial (NCT03161756) and POPCORN trial (ACTRN12618001121257)) aim to elucidate the impact of RANKL expression in the TME. However, patient selection, treatment duration, safety, dosing, and sequencing of therapies remain challenging, largely due to the lack of adequate non-invasive biomarkers for RANKL [33].

Therefore, our group previously developed [^89^Zr]Zr-DFO-denosumab as a compelling candidate imaging biomarker for RANKL expression in the TME [15]. However, slow distribution, tumor uptake, and clearance are major limitations in the clinical translation of an antibody-based radiotracer [34]. This slow blood clearance is accompanied by relatively high radiation exposure and complicates follow-up scans and translational studies. For example, a dosimetry study in patients using [^89^Zr]Zr-DFO-trastuzumab calculated values up to 30 mSv for a single PET scan [16]. The high radiation dose has been a limiting factor for clinical translation of several ^89^Zr radiolabeled antibodies. Therefore, the use of anti-RANKL Fab fragments can resolve the issues above by its lower molecular weight of 50 kDa, compared with 150 kDa (antibody), and subsequent faster renal clearance, which allows coupling with shorter-lived radioisotopes.

The anti-RANKL Fab fragment in this study is digested from denosumab to maintain identical epitope binding sites between the antibody and Fab fragment. This strategy was also selected since denosumab is a registered drug used routinely in clinical practice. The generation of Fab fragments via papain digestion is a well-described, easy-to-use method and requires minimal optimization, whereas preparing other antibody fragments (VHH, VNAR, scFv) requires extensive know-how [35]. Furthermore, it applies to multiple antibodies of different origins and subtypes [36]. Denosumab Fab fragment digestion yields were in accordance with yields in similar experiments using trastuzumab [37]. However, purification of denosumab Fab fragments proved more challenging, as protein A purification, generally the standard method, was ineffective (Figure S1). It has been reported that Fab fragments containing a V_H_3 variable domain (which is the case for denosumab) show protein A specific binding and are therefore unable to be purified from the Fc and intact antibody using this method [38,39]. Minor differences could be observed during the Kd evaluation of the antibody and Fab fragment that may be attributed to the antibody’s multivalent binding sites, leading to higher avidity—a phenomenon that has been described before [37].

To allow a head-to-head comparison of the antibody and Fab fragment, ^64^Cu has an ideal half-life (12.7 h) and was selected as the radioisotope of interest for this study. [^64^Cu]Cu-NOTA-denos-Fab and [^64^Cu]Cu-NOTA-denosumab were radiolabeled in good radiochemical yield, with good apparent specific activity and good stability. Metal complex stability was evaluated using the iTLC method, showing intact radiolabeled complex up to 40 h. However, antibody/Fab fragment integrity could not be assessed using this method. Instead, radio-SEC assays should be performed in the future. In vivo, [^64^Cu]Cu-NOTA-denos-Fab and [^64^Cu]Cu-NOTA-denosumab both show significantly higher uptake in RANKL-ME-180 (RANKL-positive) xenografts compared with ME-180 (RANKL-negative) tumors. However, [^64^Cu]Cu-NOTA-denos-Fab emerged as the best radiotracer of the two, showing better tumor permeation and higher tumor/blood ratios at earlier time points after administration. Additionally, RANKL expression in xenografts was confirmed via immunofluorescent analysis. These results support the possibility of same-day imaging and increase the feasibility of implementing the radiotracer in the clinic [40].

However, a limitation of the Fab fragment radiotracer could be the reduced absolute tumor uptake compared with the antibody (2.72 vs. 6.88% ID/mL), a phenomenon also identified in other pre-clinical studies. This observation could be due to the removal of the Fc fragment, which contributes to the internalization of the antibody. However, the Fc fragment can also result in non-specific binding and can trigger immune responses—both of which are undesirable for imaging applications [41]. Improving the targeting of the tumor could also be achieved by using a Fab2 fragment that offers a higher binding valency. However, the higher MW of a Fab2 fragment is associated with longer blood circulation times, resulting in later imaging time points [42].

Notably, compared with our previous study with [^89^Zr]Zr-DFO-denosumab, a significant reduction in bone uptake could be observed with [^64^Cu]Cu-NOTA-denos-Fab, caused by the suboptimal in vivo stability of the DFO chelator [43]. This represents a substantial improvement since anti-RANKL imaging may also have potential applications in characterizing the bone metastatic niche [44].

A limitation of the present study is that transduced cell lines do not resemble physiologically relevant amounts of RANKL expression in the tumor or its microenvironment, precluding an assessment of the biological relevance of the observed uptake. However, as the purpose of this study was to demonstrate the specificity of the radiotracer and to preliminarily characterize its biokinetic profile, our experimental setup seems appropriate. Additionally, denosumab only binds human RANKL, meaning that murine RANKL was not measured in the present study [45,46]. Therefore, additional translational pre-clinical work is needed to further evaluate the potential of [^64^Cu]Cu-NOTA-denos-Fab in more appropriate clinical models (e.g., humanized mice models or transgenic models) to study the interaction of host- and tumor-derived RANKL in the tumor microenvironment [47].

## 5. Conclusions

In conclusion, we developed [^64^Cu]Cu-NOTA-denos-Fab in good yields, with high apparent specific activity and favorable stability. The radiotracer targeted human RANKL specifically and showed improved tumor-to-background ratios compared with [^64^Cu]Cu-NOTA-denosumab. Using this strategy, single-day imaging of RANKL expression in the TME is within reach, paving the way for future clinical applications.

## Figures and Tables

**Table 1:** Bioconjugates loading efficiency and Kd determination.

Bioconjugate	NOTA-to-Fab/Antibody Ratio	Kd Value (95% Confidence Interval)
Native Fab	/	0.70 nM (0.39–1.01)
NOTA-denos-Fab (5eq)	0–3	0.31 nM (0.16–0.45)
NOTA-denos-Fab (10 eq)	1–4	0.72 nM (0.30–1.15)
Native denosumab	/	0.20 nM (0.13–0.27)
NOTA-denosumab (5 eq)	0–2	0.15 nM (0.10–0.21)
NOTA-denosumab (10 eq)	8–10	0.43 nM (0.16–0.70)

**Table 2:** Overview of radiolabeling of Fab fragments and denosumab.

Radiotracer	Non-Decay Corrected Yields (%)	Apparent Specific Activity (MBq/µg)	Radiochemical Purity (%)	Stability	
				Final Formulation (RT *)	Mouse Plasma (37 °C)
[^68^Ga]Ga-NOTA-denos-Fab	37 ± 8.7%	0.92 ± 0.2	>99%	>80% intact radiotracer for at least 2 h	
[^68^Ga]Ga-NOTA-denosumab	65 ± 5.9%	0.75 ± 0.3	>99%	>95% intact radiotracer for at least 5 h	
[^64^Cu]Cu-NOTA-denos-Fab	58 ± 9.2%	0.79 ± 0.11	>95%	>90% intact radiotracer for at least 40 h	
[^64^Cu]Cu-NOTA-denosumab	73 ± 3.5%	0.19 ± 0.02	>95%	>90% intact radiotracer for at least 40 h	

* RT—room temperature.

## Data Availability

Data are contained within the article or Supplementary Materials.

## Supplementary Materials

The following supporting information can be downloaded at: https://www.mdpi.com/article/10.3390/pharmaceutics14050939/s1, Figure S1: Protein A Fab fragment purification (A) SDS-PAGE of denosumab digestion. Lane 1: molecular weight markers; lane 2: non-reduced denosumab; lane 3: non-reduced column eluate; lane 4: non-reduced protein A column flowthrough; lane 5: non-reduced protein A column eluate (B) SEC-HPLC of protein A column flowthrough (UV signal). (C) SEC-HPLC of protein A column eluate (UV signal). (Ab = antibody, Hc = heavy chain, Lc = light chain, Fab = antibody Fab fragment, Fc = crystallizable fragment region), Figure S2: [^68^Ga]Ga-NOTA-denos-Fab cell binding—Binding to RANKL-ME-180, RANKL-ME-180-BLOCK cells of [^68^Ga]Ga-NOTA-denos-Fab at 5, 20, 50, and 100 nM radiotracer concentrations (mean ± 1 standard deviation, asterisk: statistically significant), Figure S3: Maximum intensity projections (MIP)—maximum intensity projections of [^64^Cu]Cu-NOTA-denos-Fab (left) and [^64^Cu]Cu-NOTA-denosumab (right) at 5 h post radiotracer injection in RANKL-ME-180 xenografts (p.i.: post injection, H: heart, K: kidneys, BL: bladder, T: tumor), Table S1: Yields of denosumab digestion using SpeB enzyme.
