# Supplementary material for: Improved Characteristics of RANKL Immuno-PET Imaging Using Radiolabeled Antibody Fab Fragments
Source: Pharmaceutics. 2022 Apr 26;14(5):939. doi: 10.3390/pharmaceutics14050939 (PMC9147590; doi:10.3390/pharmaceutics14050939)
Supplement: Supplementary file 1 [file pharmaceutics-14-00939-s001.zip › pharmaceutics-1653556-SI.pdf]

SUPPLEMENTARY DATA

Denosumab Fab fragment digestion: an alternative approach

In our search to evaluate the most optimal method of Fab fragment preparation, denosumab was also site specifically cleaved in the hinge region (KTHT / CPPCPAPE) using the FabULOUS® (SpeB) enzyme (Genovis, Lund, Sweden). For this, denosumab was processed as described above, and the digestion was performed using 0.5 mg denosumab, 500 units SpeB enzyme, in 50 mM cysteine PBS solution and followed up for 25 h at 37 °C under continuous stirring. The formation of Fab fragments and digestion yields were evaluated via SEC-HPLC as described above.

Digestion was successful and yields were similar as described for the papain approach (Table S1). However, the substantially higher costs of the SpeB enzyme and the more difficult removal of the enzyme (non-resin based) compared to the papain approach made this method suboptimal for our application.

Table S1: Yields of denosumab digestion using SpeB enzyme.

Incubation	% Fab & Fc fragments
1 h	37 %
2 h	49 %
5 h	71 %
25 h	87 %

Anti-RANKL-Fab fragment protein A purification

Protein A is extensively described in the literature for Fab fragment purification and was also explored in this project. For this, the protein A plus agarose NAb spin column (Thermo Fisher Scientific) was equilibrated with PBS, and subsequently, digest solution was applied to the column and incubated for 10 min at room temperature. Afterward, the column was eluted and washed two times with PBS (column flowthrough). Captured proteins were removed from the column via wash with 0.1 M glycine buffer pH 2.8 (column eluate).

The protein A column could not purify Fab fragments from other impurities as Fab fragments were also retained on the column and not present in the flowthrough, as confirmed by SDS-PAGE and SEC-HPLC (Figure S1).

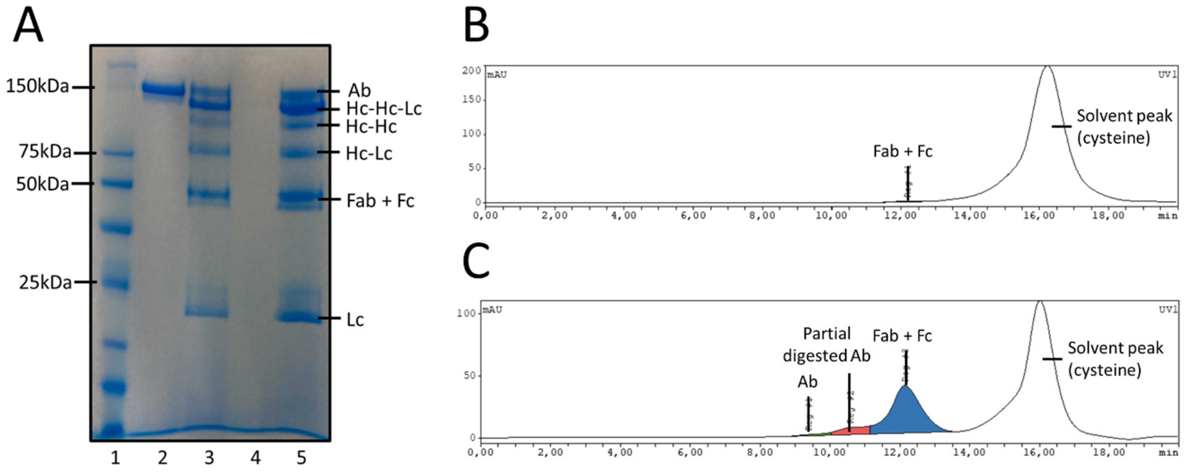

Figure S1 – *Protein A Fab fragment purification* –A) SDS-PAGE of denosumab digestion. Lane 1: molecular weight markers; lane 2: non-reduced denosumab; lane 3: non-reduced column eluate; lane 4: non-reduced protein A column flowthrough; lane 5: non-reduced protein A column eluate B) SEC-HPLC of protein A column flowthrough (UV signal). C) SEC-HPLC of protein A column eluate (UV signal). (Ab = antibody, Hc = Heavy chain, Lc = Light chain, Fab = Antibody fab fragment, Fc = crystallizable fragment region).

Cel binding: [^{68}Ga]Ga-NOTA-denos-Fab/denosumab

The initial characterization was performed with ^{68}Ga, and an *in vitro* cell binding assay was performed. In a subset of Eppendorf's, a solution of 1 x 10^6 (RANKL-ME-180, V = 0.5 mL) cells was created and incubated for 30 min at 37 °C prior to the start of the assay. [^{68}Ga]Ga-NOTA-denos-Fab was added to the Eppendorf's in a total concentration of 5, 20, 50 and 100 nM and incubated for 2 h at 37 °C. As controls in this assay, a blocking study was performed in which a molar excess of 325 x native unconjugated denosumab was added to each well and preincubated for 1 h at 37 °C. Afterward, cells were centrifuged and washed once before counting pellet and supernatants on the gamma counter. Radiotracer binding was expressed as percentage cell-bound associated activity. Significant binding to the RANKL-ME-180 expressing cells could be visualized in all radiotracer concentrations compared to the blocking dose (Figure S2).

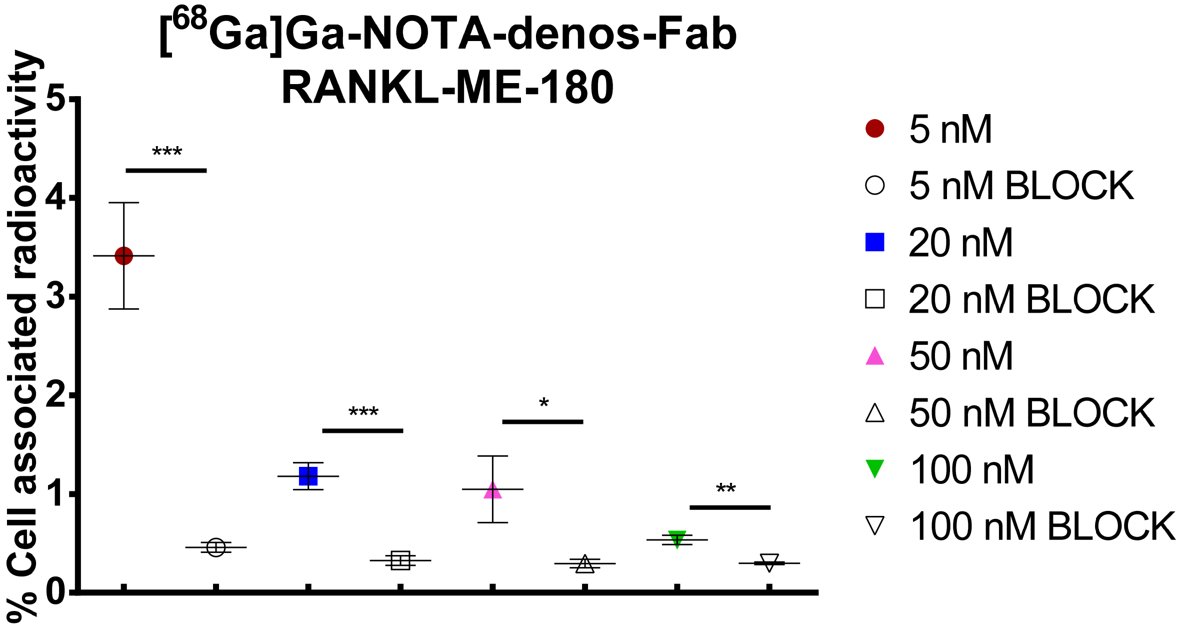

Figure S2 – *[^{68}Ga]Ga-NOTA-denos-Fab cell binding* – Binding to RANKL-ME-180, RANKL-ME-180-BLOCK cells of [^{68}Ga]Ga-NOTA-denos-Fab at 5, 20, 50 and 100 nM radiotracer concentrations (mean +/- 1 standard deviation; * $p \leq 0.05$; ** $p \leq 0.01$; *** $p \leq 0.001$).

Maximum intensity projections

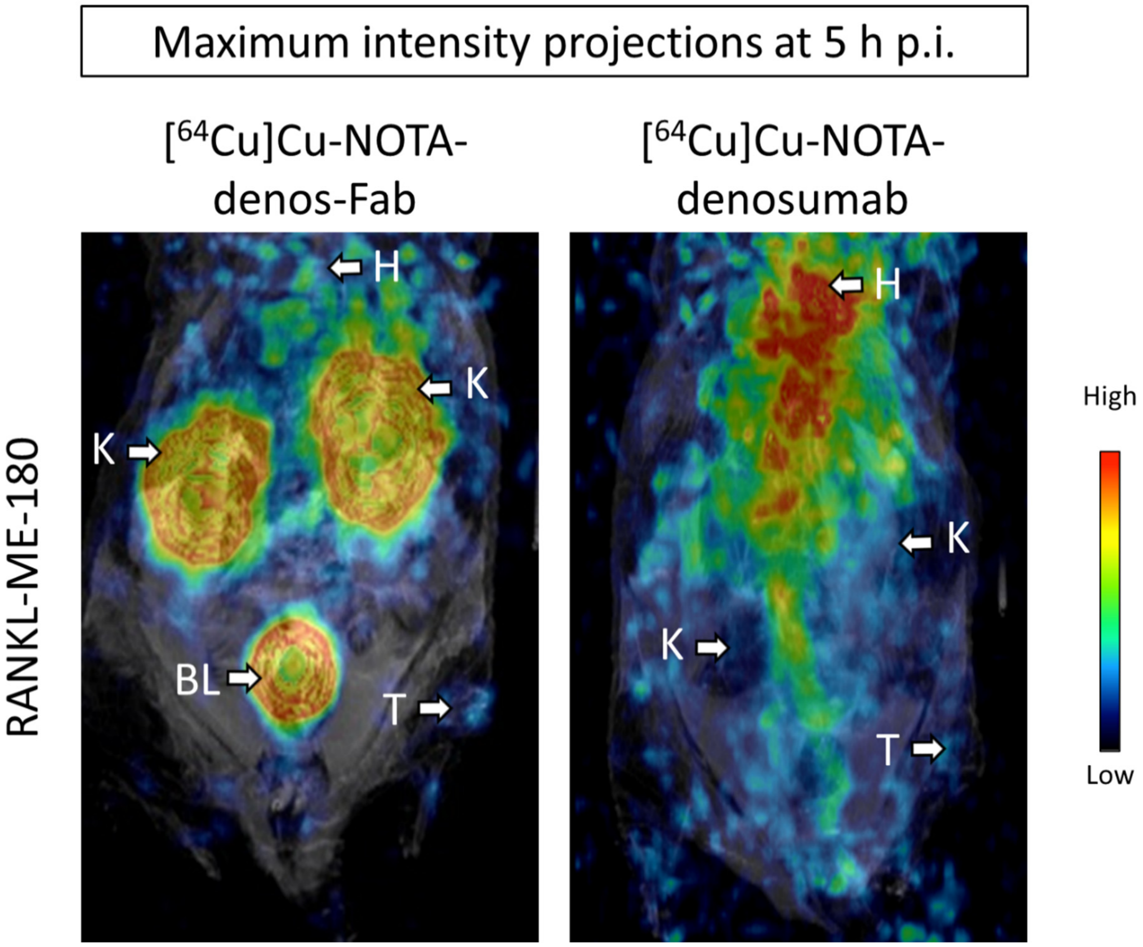

Figure S3 – *Maximum intensity projections (MIP)* – Maximum intensity projections of [^{64}Cu]Cu-NOTA-denos-Fab (left) and [^{64}Cu]Cu-NOTA-denosumab (right) at 5 h post radiotracer injection in RANKL-ME-180 xenografts (p.i.: post injection, H: Heart, K: Kidneys, BL: Bladder, T: Tumor).
